# Radiomic features of the hippocampal based on magnetic resonance imaging in the menopausal mouse model linked to neuronal damage and cognitive deficits

**DOI:** 10.1007/s11682-023-00808-z

**Published:** 2023-12-16

**Authors:** Jie Zhao, Yan Jiao, Hui Wang, Peiji Song, Zhen Gao, Xue Bing, Chunling Zhang, Aimei Ouyang, Jian Yao, Song Wang, Huijie Jiang

**Affiliations:** 1 Department of Radiology, The Second Affiliated Hospital of Harbin Medical University, Harbin, 150086 China; 2 Department of Radiology, Central Hospital Affiliated to Shandong First Medical University, Jinan, Shandong China; 3 Department of Radiology, Longhua Hospital, Shanghai University of Traditional Chinese Medicine, No.725, South Wanping Road, Shanghai, 200032 China

**Keywords:** Estrogen deficiency, MRI, Radiomics features, Ovariectomized mouse, Cognitive decline

## Abstract

Estrogen deficiency in the early postmenopausal phase is associated with an increased long-term risk of cognitive decline or dementia. Non-invasive characterization of the pathological features of the pathological hallmarks in the brain associated with postmenopausal women (PMW) could enhance patient management and the development of therapeutic strategies. Radiomics is a means to quantify the radiographic phenotype of a diseased tissue via the high-throughput extraction and mining of quantitative features from images acquired from modalities such as CT and magnetic resonance imaging (MRI). This study set out to explore the correlation between radiomics features based on MRI and pathological features of the hippocampus and cognitive function in the PMW mouse model. Ovariectomized (OVX) mice were used as PWM models. MRI scans were performed two months after surgery. The brain’s hippocampal region was manually annotated, and the radiomic features were extracted with PyRadiomics. Chemiluminescence was used to evaluate the peripheral blood estrogen level of mice, and the Morris water maze test was used to evaluate the cognitive ability of mice. Nissl staining and immunofluorescence were used to quantify neuronal damage and COX1 expression in brain sections of mice. The OVX mice exhibited marked cognitive decline, brain neuronal damage, and increased expression of mitochondrial complex IV subunit COX1, which are pathological phenomena commonly observed in the brains of AD patients, and these phenotypes were significantly correlated with radiomics features (p < 0.05, |r|>0.5), including Original_firstorder_Interquartile Range, Original_glcm_Difference Average, Original_glcm_Difference Average and Wavelet-LHH_glszm_Small Area Emphasis. Meanwhile, the above radiomics features were significantly different between the sham-operated and OVX groups (p < 0.01) and were associated with decreased serum estrogen levels (p < 0.05, |r|>0.5). This initial study indicates that the above radiomics features may have a role in the assessment of the pathology of brain damage caused by estrogen deficiency using routinely acquired structural MR images.

## Introduction

The secretion of estrogen rapidly declines post-menopause and leaves postmenopausal women (PMW) at higher risk of various physical and mental illnesses than men. The incidence of neurodegenerative disease in females is higher than in males. Clinical studies have shown that women are more likely than men to develop mild cognitive impairment and Alzheimer’s disease (AD) (Mulnard et al., 2000). The cognitive function of postmenopausal women will show the degeneration of the central nervous system with the aging of their ovarian function, and the most prominent and earliest manifestation is mainly the reduction of memory function (Gresack & Frick, 2004; Nelson et al., 2012). Extensive evidence from animal studies indicates that the hormone disturbance caused can lead to cognitive decline in ovariectomized (OVX) female mice, and the pathological staining of brain sections shows that neurons in the hippocampus are damaged (Chou et al., 2015; Krolick et al., 2018). At present, there is sufficient evidence that the imbalance of brain metabolism caused by hormonal disorders is an important reason why menopausal women are more susceptible to AD. Similar data were obtained from the brains of patients with AD. Namely, an increased expression of mitochondrial complex IV subunits (COX-1) and an increased level of mitochondrial DNA was detected in the hippocampus and cortex of patients with sporadic AD (Kodama & Gan, 2019; Park et al., 2018; Slopien et al., 2018).

Over the last 30 years, magnetic resonance imaging (MRI) has become an important tool in clinical diagnostics and basic neuroscience research (Chandra et al., 2019). The latest advancements in cognitive science point out that distinct activities of the brain might be recognized using neuroimaging data. Advances in neuroimaging provide unique opportunities to evaluate brain structure, biochemistry, and function (Cai et al., 2020; Du et al., 2020). Additionally, Magnetic resonance imaging (MRI)-based radiomics has been used to derive heterogeneity information in cerebral tissue by extracting radiomics features from structural MRI in AD as well as other neurological disorders. Brain structures assessed by neuroimaging can be semantically enriched by available molecular biological data such as neural receptor densities or gene expression in this brain area (Lee et al., 2020; Lombardi et al., 2020; Vamvakas et al., 2022). At present, radiomics as an emergent imaging analysis is widely used in the imaging studies of neurodegenerative disease diagnosis, post-treatment, and prognosis evaluation. Through the use of radiomics, quantitative radiographic phenotype features (intensity, shape, texture, and transformed features) can be extracted from multiple medical image modalities. Radiomics involves the high-throughput extraction of a large number of quantitative characteristics from medical imaging, which can provide complete information on brain tissue radiophenotype and microenvironment heterogeneity (Yi et al., 2021; Nowakowski et al., 2022). It is currently unknown, however precisely how these radiomics features relate to cognitive decline and associated pathologies in PMW.

In this study, we applied radiomics analysis to T2 weighted structural MRI scans of an animal model of PMW, the OVX mouse model of estrogen-deficient, and litter-matched sham-operated mice. In this study, we used pyradiomics to extract texture features, an open-source python package for extracting radiomics features from medical imaging. This is a reference standard for Radiomic Analysis, and provides a tested and maintained open-source platform for easy and reproducible Radiomic Feature extraction. The platform supports both feature extraction in 2D and 3D and can be used to calculate single values per feature for a region of interest (segment-based) or to generate feature maps (voxel-based) (Du et al., 2021; He et al., 2021). Many research projects have been developed to perform pathology-related analyses using features extracted from medical images. radiomics analysis can show subtle structural changes in tissues that are not visible on MR images (Humans can only discern approximately 80 different gray levels in the image) (Ni, 2021; Xue et al., 2021; Nguyen et al., 2012). As such the changes in the heterogeneity of tissue detected by radiomics analysis could reflect the brain damage caused by estrogen deficiency.

The purpose of this study was (i) to explore the difference in the features between the PMW mouse model and control groups, and (ii) to explore the correlation between radiomics features in the PMW mouse model with the peripheral blood estrogen levels, cognitive performance, the extent of neuronal damage, and the expression of COX-1.

## Materials and methods

### Animals

Female C57/BL6 mice (9-week-old) were purchased from Weitong Lihua Experimental Animal Central (Beijing, China). The animal rooms were maintained with a temperature of 24 °C, relative humidity of 40–70%, and a light/dark cycle of 12 h. Surgery was performed after 1 week of transportation to adapt to the environment. The animal experiment strictly adhered to principles to minimize the experimental animals’ use, pain, and suffering.

### Surgery

The 10-weeks-old mice were bilaterally ovariectomized (OVX group, n = 31) or sham-operated (Sham group, n = 30) after being anesthetized with pentobarbital sodium (50 mg/kg) administered intraperitoneally. The wound was sutured and the animals were returned to their cages. Further experiments were performed after two months of surgery.

### Morris water maze test

Morris’s water maze test was performed as described, including an orientation navigation experiment during the first five days and the probe trial on the sixth day (Bittar et al., 2022). The maze is comprised of a circular pool 150 cm in diameter and 20 °C water temperature. The water maze was divided into four quadrants with the hidden platform located in the center of one target quadrant. The time of the mice to find the hidden platform was recorded as the escape latency time. On the sixth day of the test, a probe trial was conducted without the platform. The shorter the escape latency, the better their spatial learning and memory ability. Mice were recorded with digital video cameras (Logitech C920) and behavioral indices were measured using an automated video-tracking system (ANY-maze).

### MRI

All MRI measurements were done on a 9.4T small animal MRI scanner (BioSpec 94/20 USR, Bruker Biospin). Before MRI scanning, animals were anesthetized by inhalation of isoflurane (4%, RWD Life Science, Shenzhen, China) and maintained under isoflurane (1.5%) narcosis during MRI scanning. Core body temperature and respiration were monitored during scanning (SA instruments). RF transmission was performed with a 72 mm volume coil and a 4-channel receiver coil (Rapid Biomedical). The following parameters were used for T2WI: Matrix size = 512 × 512; FOV = 19.2 mm × 19.2 mm; slice number = 26; TR = 1500 ms; TE = 6.5 ms; the number of averages = 3; resolution = 37.5 μm × 37.5 μm × 250 μm; fip angle = 60°.

### Radiomics features extraction and selection

All processing was done using the 3D-Slicer software tool (3D Slicer, Version 4.11.0). N4 bias field correction was applied to each sequence image to correct intensity non-uniformities. The region of interest (ROI) was manually placed by the same radiologist across all contiguous slices on which the hippocampus was visible via a hand annotation tool in 3D-Slicer software. Feature extraction was based on the platform and performed using the pyradiomics package (http://PyRadiomics.readthedocs.io/en/latest/), which can extract 1026 parameters, including shape features, first-order features, gray level co-occurrence matrix, gray-level run-length matrix, etc. For dimensionality reduction and to avoid over fitting, we the least absolute shrinkage and selection operator (LASSO) regression algorithm to choose the most significant radiomic features based on minimum criteria. LASSO regression was performed using the glmnet (Lasso and Elastic-Net Regularized Generalized Linear Models) package, version 2.0–2, in the R statistical package (version 3.0.3). Finally, we selected 11 radiomic features for study (Table 1).

**Table 1:** Comparison of radiomic features of Mr. T2WI images between sham group and OVX group

Image type	Feature Class	Feature [Median (IQR)]	Sham (n = 30)	OVX (n = 31)	p
Original	firstorder	Interquartile Range	5.524[5.376,5.644]	7.445[7.311,7.847]	< 0.001
Original	glcm	Difference Average	0.168[0.149,0.204]	0.297[0.259,0.363]	0.001
Original	glcm	Sum Average	-0.722[-0.894,-0.380]	-0.785[-1.321,-0.336]	0.75
Original	glrlm	Run Variance	9.380[8.803,9.683]	5.547[5.385,6.446]	< 0.001
Log-sigma	glcm	Joint Energy	9.637[5.026,15.369]	9.730[4.441,78.662]	0.534
Log-sigma-3	glrlm	Gray Level	0.079[0.070,0.117]	0.087[0.033,0.122]	0.796
log-sigma-5	glrlm	Gray Level	0.015[0.013,0.034]	0.018[0.003,0.035]	0.727
Wavelet-LLH	gldm	Dependence Entropy	0.152[0.126,0.270]	0.130[0.046,0.297]	0.242
Wavelet-LHL	firstorder	Skewness	38.202[35.331,53.287]	38.190[24.713,84.917]	0.867
Wavelet-LHH	glszm	Small Area Emphasis	1.572[1.180,2.373]	5.367[4.624,6.847]	< 0.001
Wavelet-LLL	glszm	Zone Entropy	154.416[131.664,221.000]	144.407[92.000,341.000]	0.761

Radiomic features were described using the median [IQR: interquartile range]

### Tissue collection and estrogen measurement

After euthanasia, blood was also collected by cardiac puncture and centrifuged to separate serum. Serum concentrations of estradiol were measured using chemiluminescence (Beckman Coulter). The brain tissues were removed immediately and immersed in 4% paraformaldehyde for 24 h.

### Hematoxylin and eosin (HE) staining

Paraffin-embedded brains were sectioned to a thickness of 5 μm. HE staining was conducted according to routine protocols with a HE staining kit (Solarbio, Beijing, China). Briefly, tissue sections underwent dewaxing and rehydration through xylene and ethanol treatment and were subsequently stained with Harris’s hematoxylin for 3 min, washed with distilled water, and then put into filtered eosin for 30 s.

### Nissl staining

As previously described, the relative healthy state of hippocampal neurons was evaluated using Nissl staining. The sections were stained with 1% Cresyl Violet (C5042, Sigma-Aldrich) and covered with 50% glycerin. Cells were counted on three randomly selected non-overlapping fields in each slide of the hippocampal tissue with the proportion of healthy cells defined as the number of Nissl positive cells /total number of cells. The sectioned tissues were scanned with a confocal microscope (LSM 510 META; Carl Zeiss).

### Immunostaining

The immunohistochemical experiment was carried out as mentioned earlier (Li et al., 2021). The antibodies used in this study are as follows: anti-COX-1 antibody (1:50; ab109025; Abcam), anti-NeuN antibody (1:200; ab104224; Abcam), Alexa Fluor 488 secondary antibody (1:1,000; Invitrogen), Alexa Fluor 594 secondary antibody (1:1,000; Invitrogen). Cell nuclei were stained with DAPI (Sigma-Aldrich). Images were captured LSM700 laser scanning confocal microscope (Zeiss). Mean fluorescence intensities (MFI) were determined using FlowJo software.

### Statistical analysis

Variables were tested with Mann-Whitney U tests or Student’s t-tests depending on the distribution of the data, as appropriate, and a two-sided p-value < 0.05 was considered statistically significant. If the data were normally distributed, then Pearson correlation was used for quantifying correlation; otherwise, the Spearman correlation coefficient was used. All statistical analyses were performed using R statistical software version 3.5.3.

## Results

### OVX caused cognitive impairment and neuronal injury in the hippocampus of mice

**Fig. 1:**
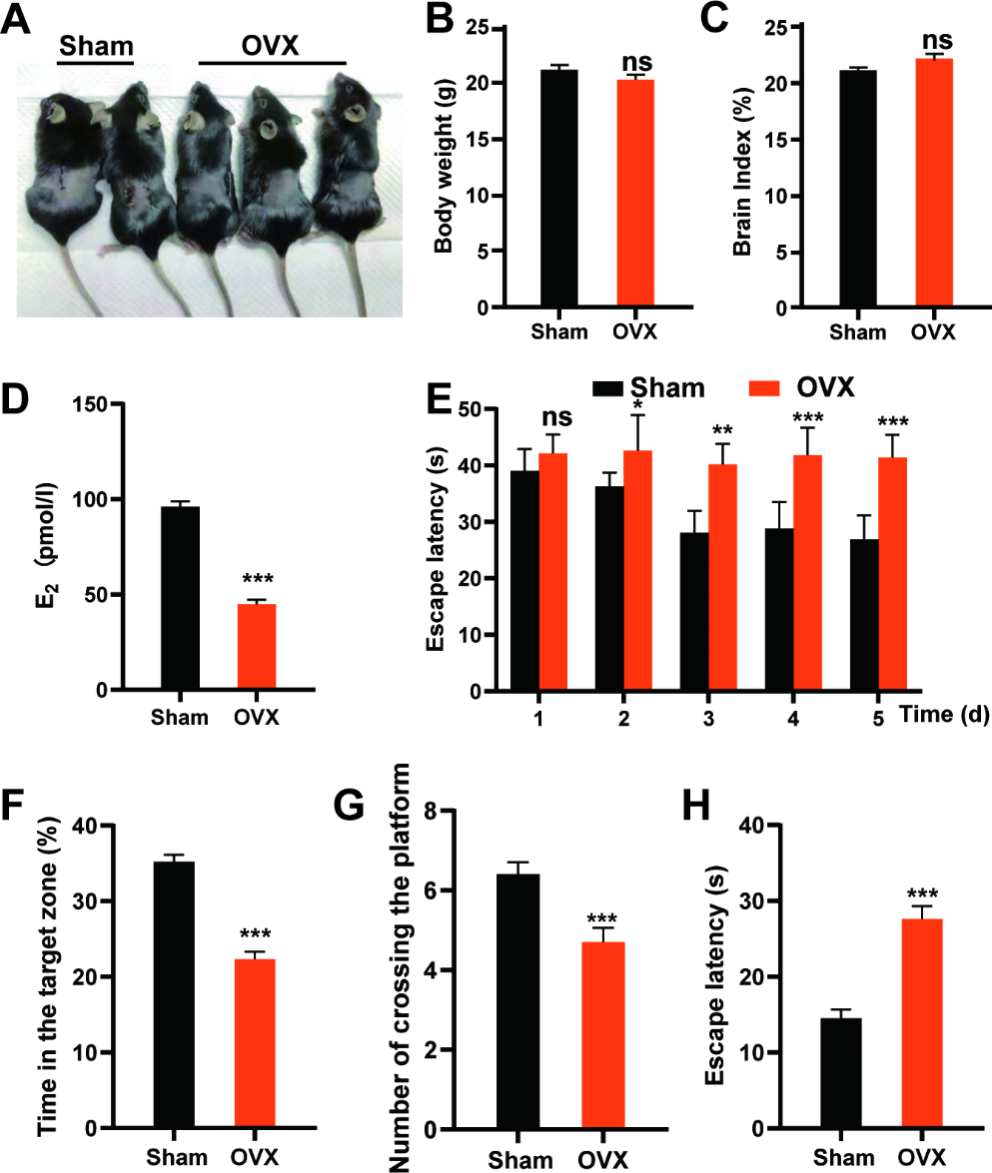
Ovariectomy (OVX) caused estrogen deficiency and cognitive impairment in mice. (**A**) Image of female C57/BL6 mice after sham surgery or OVX. Body weights (**B**) and cerebral index (**C**) of mice in each group at 2 months postoperatively. Data represent the mean ± standard error of the mean. ns: no statistical significance. In each group at least 10 mice were tested. (**D**) Serum estrogen levels in the two study groups after ovariectomy or sham operations. (**E**) Escape latency in the positioning navigation experiment for each group. (**F**) Percentage of total time spent in the target quadrant during the probe trials in each group. (**G**) The number of times that the mice crossed the platform in each group in the probe trials. (**H**) Escape time to target each group in the probe trials. Data represent the mean ± standard error of the mean. Student’s t-test: *p < 0.05, **p < 0.01 and ***p < 0.001. ns: no statistical significance. In each group, at least 10 mice were tested

In this study, the body and brain of mice were weighed, and the brain index (brain weight/body weight) values were calculated at 2 months postoperatively (Fig. 1A-C). Surgical ovariectomy produced no significant change in body weight (Fig. 1B) and the brain index (Fig. 1C) in the OVX group, as compared to the Sham group. These results suggest that different surgical procedures do not significantly affect the development of mice, in parallel, the body weight and brain index should not be a factor in our subsequent studies.

Estrogen deficiency is associated with the OVX-mediated cognitive deficit (Huebner et al., 2022). Similar to previous theoretical studies, serum estrogen levels in the OVX group were significantly lower than those in the Sham group (Fig. 1D).

Morris water maze was used to evaluate the learning and memory ability of mice in each group. Results of the directional navigation experiments for five consecutive days demonstrated that OVX mice showed significantly higher escape latencies than those of Sham mice (Fig. 1E). Moreover, the results of the probe trials on the 6th day showed that the OVX group spent less time in the target quadrant than the Sham group (Fig. 1F). Meanwhile, the number of times that OVX mice crossed the region where the platform used to be located was significantly less than that of Sham mice (Fig. 1G). And, the time to the hidden platform in the OVX group was significantly higher than that in the Sham group (Fig. 1H). These results suggest that OVX mice have a memory impairment.

**Fig. 2:**
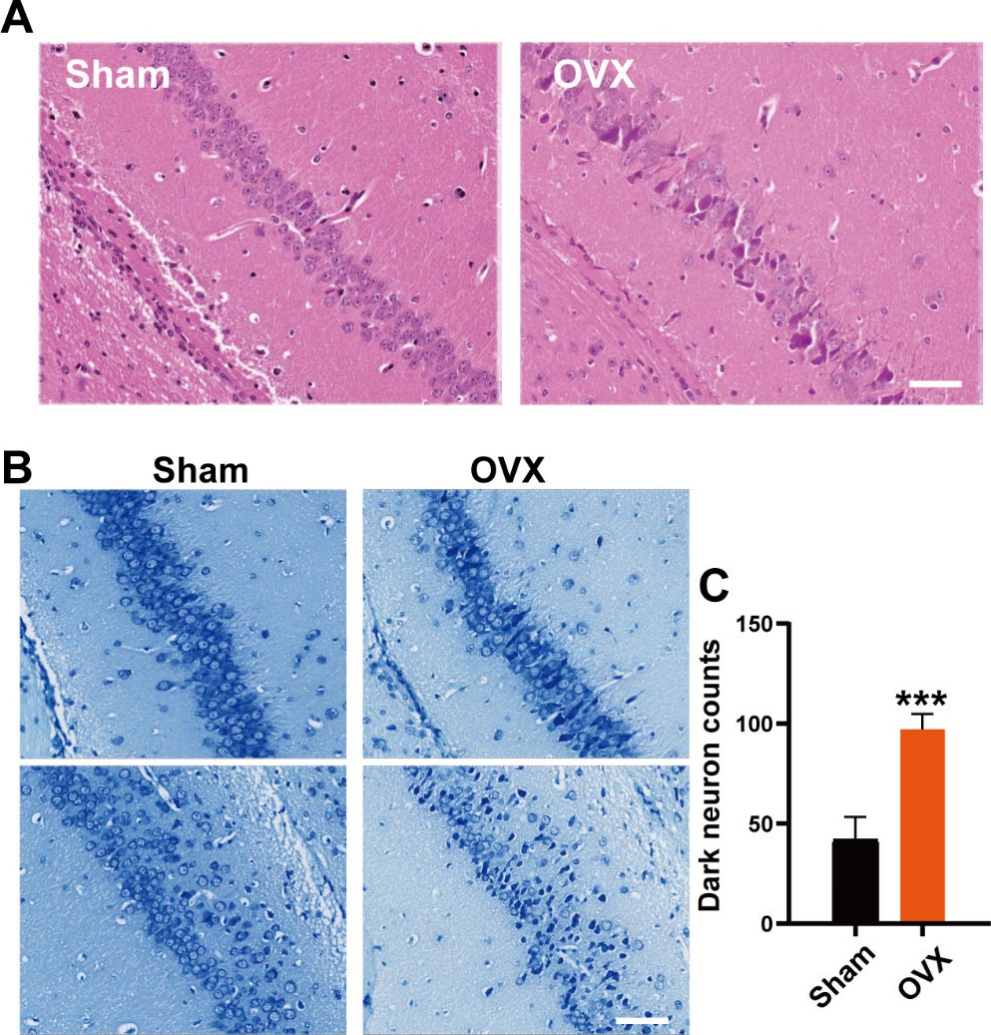
Ovariectomy caused neuronal injury in the hippocampus of mice. (**A**) Photomicrographs of H&E–stained hippocampus sections for each group of mice (bar = 50 μm). (**B**) Identification of neuronal survival by Nissl staining in the hippocampus (bar = 20 μm). (**C**) The quantity of healthy neuronal cells in each visual field with densely stained Nissl bodies. Data represent the mean ± standard error of the mean. Student’s t-test: ***p < 0.001. ns: no statistical significance. In each group, at least 10 mice were tested

The hippocampus is the key area for cognition and memory in mammals. HE staining revealed that neurons in the hippocampal CA1 region of the OVX group were arranged in a disordered, distracted pattern with blurred boundaries, compared to the Sham group (Fig. 2A). Meanwhile, the losses of Nissl-positive viable neuronal cells with typical neuropathological change were observed in the OVX group based on Nissl staining (Fig. 2B-C).

**Fig. 3:**
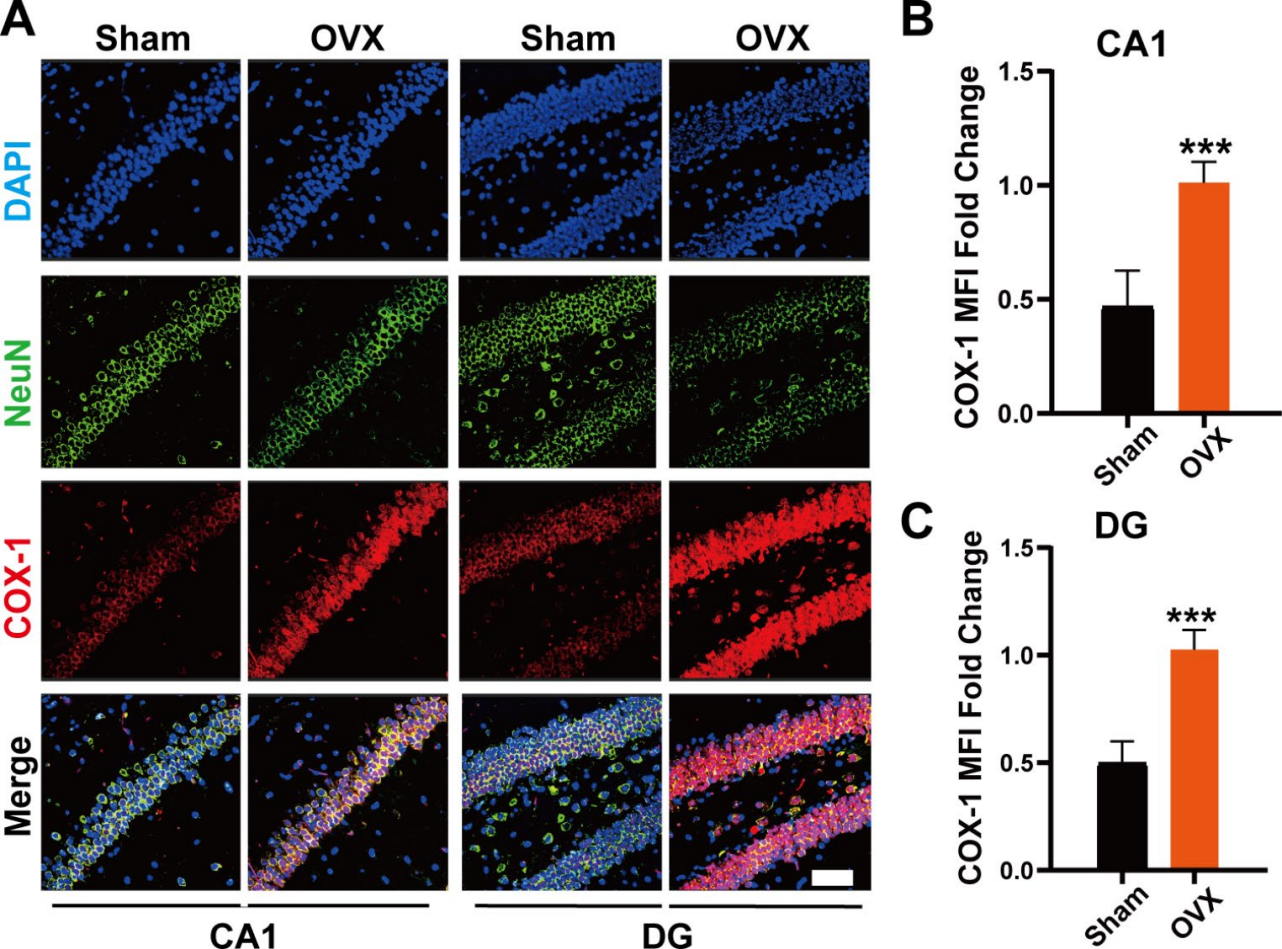
Ovariectomy increased COX-1 expression in the hippocampus of mice brains. (**A**) Typical images of neuronal cells (NeuN labeled) and COX-1 were co-located by immunofluorescence. Bar = 50 μm. (**B**) The mean fluorescence intensity of COX-1 was determined, and the levels of COX-1 intensity were expressed as a relative change in comparison with the OVX group, which was set to 100%. Data represent the mean ± standard error of the mean. Student’s t-test: ***p < 0.001. ns: no statistical significance. In each group, at least 10 mice were tested

In addition, immunofluorescence analysis showed that compared with the Sham group, the expression of COX-1 in the CA1 and DG regions of the hippocampus in the OVX group was significantly increased (Fig. 3).

### Radiomics features were significantly different among the Sham and OVX group

**Fig. 4:**
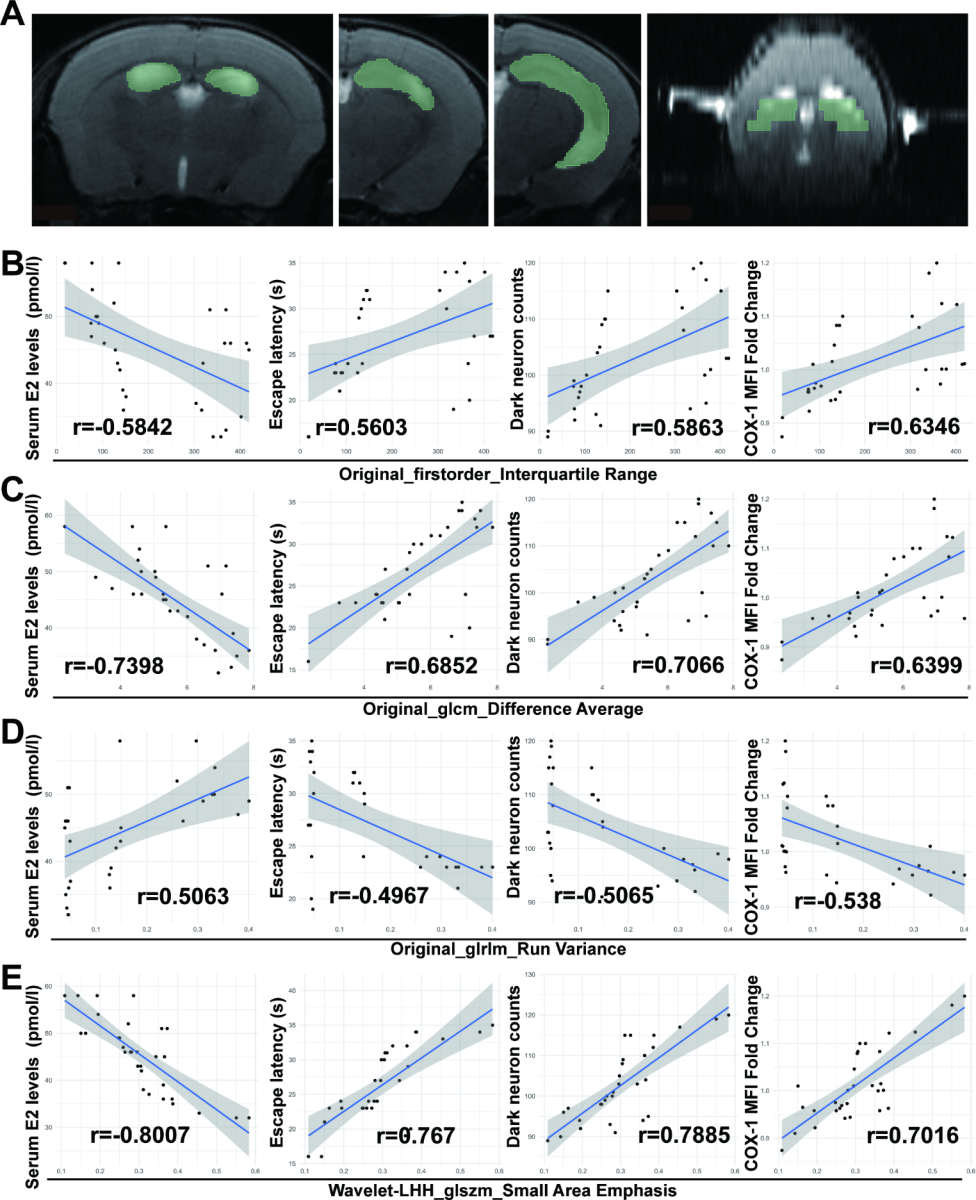
In the brain of OVX mice, radiomics features including Original_firstorder_Interquartile Range, Original_glcm_Difference Average, Original_glcm_Difference Average, and Wavelet-LHH_glszm_Small Area Emphasis, were associated with the peripheral blood estrogen levels, cognitive performance, the extent of neuronal damage, and the expression of COX1. (**A**) Mouse brain MR images: coronal and axial slices. Region of interest (ROIs) in the hippocampus was manually drawn to be analyzed with texture analysis methods. (**B-E**) Correlation curve fitting diagram (n = 31) of the radiometrics features with the peripheral blood estrogen (E2) levels (pmol/L) escape latency (s) of mice in the hidden-platform test of the Morris water maze (markers for cognitive performance), dark neurons number (biomarkers for neuronal damage), and the expression of COX1 (markers of disordered energy metabolism). The Pearson correlation and Spearman rank correlation test were used for the correlations

Considering the pathological staining results, ROIs were selected on the MR images in the hippocampus to be analyzed by radiomics analysis (Fig. 4A). Each ROI in the Sham and OVX groups was then characterized and compared using 11 radiomics features obtained by screening. The results showed that there were 4 radiomics features, which including Original_firstorder_Interquartile Range, Original_glcm_Difference Average, Original_glrlm_Run Variance, and Wavelet-LHH_glszm_Small Area Emphasis, were statistically different between the two groups (p < 0.05, Table 1). Specifically, the radiomics feature Original_firstorder_Interquartile Range, Original_glcm_Difference Average, and Wavelet-LHH_glszm_Small Area Emphasis increased while the Original_glrlm_Run Variance decreased in the OVX group compared with the sham group.

### MR radiomics features were correlated with the serum estrogen levels, behavioral and pathological indicators

To explore the relationship between the variation of MRI radiomics features induced by OVX and physiological or pathological indicators including serum estrogen level, escape latency in the probe trials, the dark neuron counts, and the expression of COX1 in the hippocampus, we conducted a correlation analysis of these radiomics features and the physiological or pathological indicators in the OVX groups separately (Fig. 4B-E).

Then, results from relevant correlation analyses indicated a significant association between those radiomics features and pathological conditions of the brain in OVX mice (Fig. 4B-E, p < 0.05, |r| > 0.5). Specifically, except for Original_glcm_Difference Average, all other features were significantly negatively correlated with serum estrogen levels (p < 0.05, r < 0). Notably, compared with other radiomics features, Wavelet-LHH_glszm_Small Area Emphasis was most correlated with cognitive decline, black neuron count, and COX-1 expression level (Fig. 4E, p < 0.001, |r|>0.7).

The above results demonstrated that the MRI-T2-based radiological features of the hippocampus of the brain caused by menopause may reflect pathological changes such as neuronal damage.

## Discussion

Estrogen is a fundamental regulator of the metabolic system of the female brain and body. During menopause, the decline in circulating estrogen is coincident with the decline in brain bioenergetics and a shift towards a metabolically compromised phenotype. Compensatory bioenergetic adaptations, or lack thereof, to estrogen loss could determine the risk of late-onset Alzheimer’s disease (Rettberg et al., 2014). OVX mice are the classic animal model of female menopause. The loss of ovariectomized mice causes a decrease in circulating estrogen, which induces brain damage (Cheon et al., 2017). In this study, we applied radiomics analysis to a mouse model of OVX and investigated its ability to noninvasively detect the pathology of brain injury induced by estrogen deficiency in this mouse model compared with control animals undergoing sham surgery. And, we sought to determine whether any radiomics features were specifically associated with serum estrogen levels, cognitive performance, neuronal damage, and the expression of COX-1 in the OVX mice.

First, we surgically removed the ovaries of female mice to establish a menopausal mouse model. When tested, peripheral blood estrogen levels in the sham mice were nearly double those in the OVX group as documented in previous studies(Jett et al., 2022; Xiong et al., 2022). Similarly, behavioral results showed a significant cognitive decline in OVX mice. Next, HE and Nissl staining showed that the OVX mice had severe neuronal damage in the hippocampal area, which is a key area for cognition and memory. Therefore, we decided to conduct a radiomics analysis of the mouse brain hippocampus based on raw MRI images, in an attempt to discover the correlation between texture parameters in this region and the pathological phenotypes related to the OVX mouse brain.

The results showed that there were significant differences in various radiomics features, including Original_firstorder_Interquartile Range, Original_glcm_Difference Average, Original_glrlm_Run Variance, and Wavelet-LHH_glszm_Small Area Emphasis, between the OVX group and the sham group. Radiomics features contain first-, second- and higher-order statistics (Reuzé et al., 2018). Specifically, Original_firstorder_Interquartile Range is a first-order feature based on the original image, which responds to the variation of the local intensity distribution of the measured voxels; Original_glcm_Difference Average, a second-order feature based on the original image, which responds to the probability of the occurrence of voxel values reflecting the given direction and distance. Original_glrlm_Run Variance describes the length of consecutive occurrences of voxels of the same grey level in a given direction; Wavelet-LHH_glszm_Small Area Emphasis is a higher order feature based on the filter that quantifies the area of consecutive voxel values in the image.

Radiomics features based on intensity, shape, size or volume, and texture provide information about the histopathological phenotype and the expression of specific proteins (Liu et al., 2016). The correlation between the imaging features and tissue pathological features has been studied for a long time. Previously, quantitative radiomics show that these imaging features correlate with the underlying gene expression profile of the tumor and can also predict the molecular typing of some tumors (Zhang et al., 2018; Li et al., 2016). Therefore, the differences in radiomics features might be related to various pathological phenotypes, requiring further investigation.

According to the results of correlation analysis, there is a strong correlation between radiomics parameters and serum estrogen levels, cognitive performance, and neuronal damage in OVX or sham mice. It should be noted that not all texture parameters were associated with physiological or pathological indicators of mice, and the combination of variables that were associated differed between the OVX and Sham groups. For example, parameters associated with estrogen levels in the sham group were not associated with estrogen levels in the OVX group. The reason for this disparate result is that the two samples are not homogenous. Specifically, since the hippocampal region of the brain is very sensitive to estrogen, the changes in related brain injury indicators in the Sham group were mainly affected by estrogen (Kim et al., 2022). In the OVX group, however, it wasn’t only estrogen deficiency that caused brain damage. Recent studies have shown that in addition to estrogen deficiency, increased follicle-stimulating hormone (FSH) and luteinizing hormone (LH) during menopause are also risk factors for the development of Alzheimer’s disease in older women (Flores et al., 2022; Harrington et al., 2022; Perich & Ussher, 2021). Therefore, the correlation between these radiomics features and other physiological hormones is also worth investigating.

In addition, we found that these radiomics features were correlated with COX-1 expression (Yang et al., 2022). Cox is a chain in the mitochondrial respiratory process, which generates ATP by oxidative phosphorylation (Wang et al., 2022). COX-1 and COX-2 are the two recognized isoforms of COX. Although COX-2 plays a much larger role than COX-1 in peripheral inflammation, COX-1 is considered to be a key contributor to neuroinflammation and energy metabolism disorders. COX-1 is constitutively expressed in most tissues and organs, including the brain(Chagas et al., 2022; Sadjjadi et al., 2019; Zhang et al., 2020). Monitoring COX-1 expression by non-invasive means is of unique significance for reflecting neuroinflammation and brain energy metabolism.

Menopausal hormonal disorders have a wide range of effects on the brain, including abnormal deposition of Aβ, hyperphosphorylation of tau protein, and other dementia pathologies, as well as increased risk of depression and mood disorders (Lombardi et al., 2020; Shu et al., 2021; Yang et al., 2020; Zhou et al., 2019). The correlation analysis of the pathological changes and imaging data and the establishing of a suitable prediction model will help us effectively monitor and intervene in the menopausal brain in clinical practice. Much remains to be done to build on this starting point.

### Limitations

First, there is structural and compositional heterogeneity between human and animal brains (Wang et al., 2021; Yang et al., 2021). Therefore, the disease-relevant radiomics features selected by mouse models should be validated in humans. Second, the effects of hormonal disturbances of menopause on the brain are widespread and not limited to the hippocampal region (Cheung et al., 2022; Harrington et al., 2022). Further radiological analysis of other brain regions including the cortex, thalamus, and cerebellum is warranted. And, we did not attempt to develop regression models to predict climacteric-related diseases using important radiological features. Finally, Such studies require large sample sizes and/or effect sizes to achieve adequate power. long-term experimental observation of large samples is lacking in this study, and underpowered by low sample size and lack of study power calculation.

## Conclusions

In conclusion, our findings suggest that radiomics features, including Original_firstorder_Interquartile Range, Original_glcm_Difference Average, Original_glcm_Difference Average, and Wavelet-LHH_glszm_Small Area Emphasis are associated with brain function and pathological status in mice. Retrospective characterization of brain degeneration from structural MRI scans will be a valuable tool for monitoring menopausal brain injury and the effectiveness of treatment strategies.

## Data Availability

Not applicable.
